# Altered methylation of glucosylceramide synthase promoter regulates its expression and associates with acquired multidrug resistance in invasive ductal breast cancer

**DOI:** 10.18632/oncotarget.9337

**Published:** 2016-05-13

**Authors:** Jiannan Liu, Xiaofang Zhang, Aina Liu, Daoping Zhang, Yi Su, Ying Liu, Dong You, Leilei Yuan, Xiangshuo Kong, Xiaodan Wang, Ping Sun

**Affiliations:** ^1^ Department of Oncology, Yuhuangding Hospital, Yantai, Shandong, 264000, P. R. China; ^2^ Department of Pathology, Shandong University School of Medicine, Jinan, Shandong, 250012, P. R. China; ^3^ Department of Rehabilitation, Qianfoshan Hospital, Jinan, Shandong, 250014, P. R. China; ^4^ Department of Radiology, Taian Central Hospital, Taian, Shandong, 271000, P. R. China

**Keywords:** glucosylceramide synthase, DNA methylation, 5-Aza-dc, breast cancer

## Abstract

Overexpression of glucosylceramide synthase (*GCS*) increases multidrug resistance (MDR) in many cancer cells. However, its mechanism is unknown. The aim of the present study is to detect the association of methylation at the *GCS* gene promoter with its expression and MDR in invasive ductal breast cancer. 40 cases *GCS*-positive and 40 cases *GCS*-negative primary breast carcinoma samples, three drug-sensitive breast cancer cell lines and one multidrug-resistant breast cancer cell line were used. Immunohistochemistry, methylation-specific PCR (MSP), quantitative real-time (qPCR), westernblot and cytotoxicity assay techniques were employed. Thwe results revealed that there was a statistically negative correlation between *GCS* CpG islands methylation and *GCS* phenotype in patients with breast cancer. *GCS* CpG islands methylation was negatively associated with high ER, meanwhile positively with high HER-2 status. Similar results were obtained from the analysis of breast cancer cell lines. Treatment with the demethylating agent 5-aza-2′-deoxycytidine (5-Aza-dc) changed the *GCS* promoter methylation pattern in three sensitive cells and also caused increased drug resistance of them. These results suggested that the changes of DNA methylation status of the *GCS* promoter correlates with multidrug resistance in breast cancer.

## INTRODUCTION

Breast cancer, the leading cause of death among women in most countries worldwide, is rapidly increasing in China [1, 2]. Despite the development of novel treatment strategies for some malignances, chemotherapy continues to be the standard therapy for most human cancers. Multidrug resistance (MDR) remains to be a serious obstacle in breast cancer treatment [3, 4]. Recently, accumulating evidence has indicated the important role of glucosylceramide synthase (*GCS*) in MDR [5, 6]. *GCS* is a transmembrane protein encoded by the UGCG gene in humans. It can transfer UDP-glucose to ceramide to form glucosylceramide, and allow cells to escape from ceramide-induced cellular apoptosis [7, 8]. Liu et al. introduced *GCS* cDNA into MCF-7 cells, which increased *GCS* enzymatic activity and resulted in resistance to doxorubicin [9]. A number of methods that suppress the expression of *GCS*, such as specific inhibitors, antisense oligonucleotides and short interfering RNA, render MDR cells chemosensitive [10, 11]. Hence, understanding of the mechanism of *GCS* expression in breast cancer cells is essential to discover novel chemotherapy targets and improve the efficacy of chemotherapy treatment.

Genetic abnormalities are insufficient to explain the mechanism of carcinogenesis. Epigenetics is becoming an important field of cancer research. DNA methylation is the predominant epigenetic modification that inhibits gene expression [12]. Mammalian DNA is heavily methylated at cytosine residues within CpG dinucleotides, with 60–80% of such residues being methylated [13]. Various genes show an inverse relationship between DNA methylation and transcription in normal and malignant cells [14]. Growing evidence indicates that DNA methylation status might be involved in MDR. The *MDR1* promoter contains a CpG island that may be inhibited by methylation [15, 16, 17]. The breast cancer resistant protein (*BCRP*) has a promoter with the similar CpG island that has been shown to inhibit gene expression via methylation [18]. The human *GCS* protein is a glycoprotein containing 394 amino acids encoded by 1182 nucleotides. *GCS* includes a G + C rich 5′ untranslated region of 290 nucleotides, containing a CpG island [19]. These findings suggested that DNA methylation might also be involved in inhibiting *GCS* expression. No research has determined the role of DNA methylation in the transcriptional regulation of *GCS* in breast cancer cells. This study aimed to rectify this omission from the literature.

## RESULTS

### *GCS* promoter methylation associates with its expression and clinicopathological parameters

**Figure 1:**
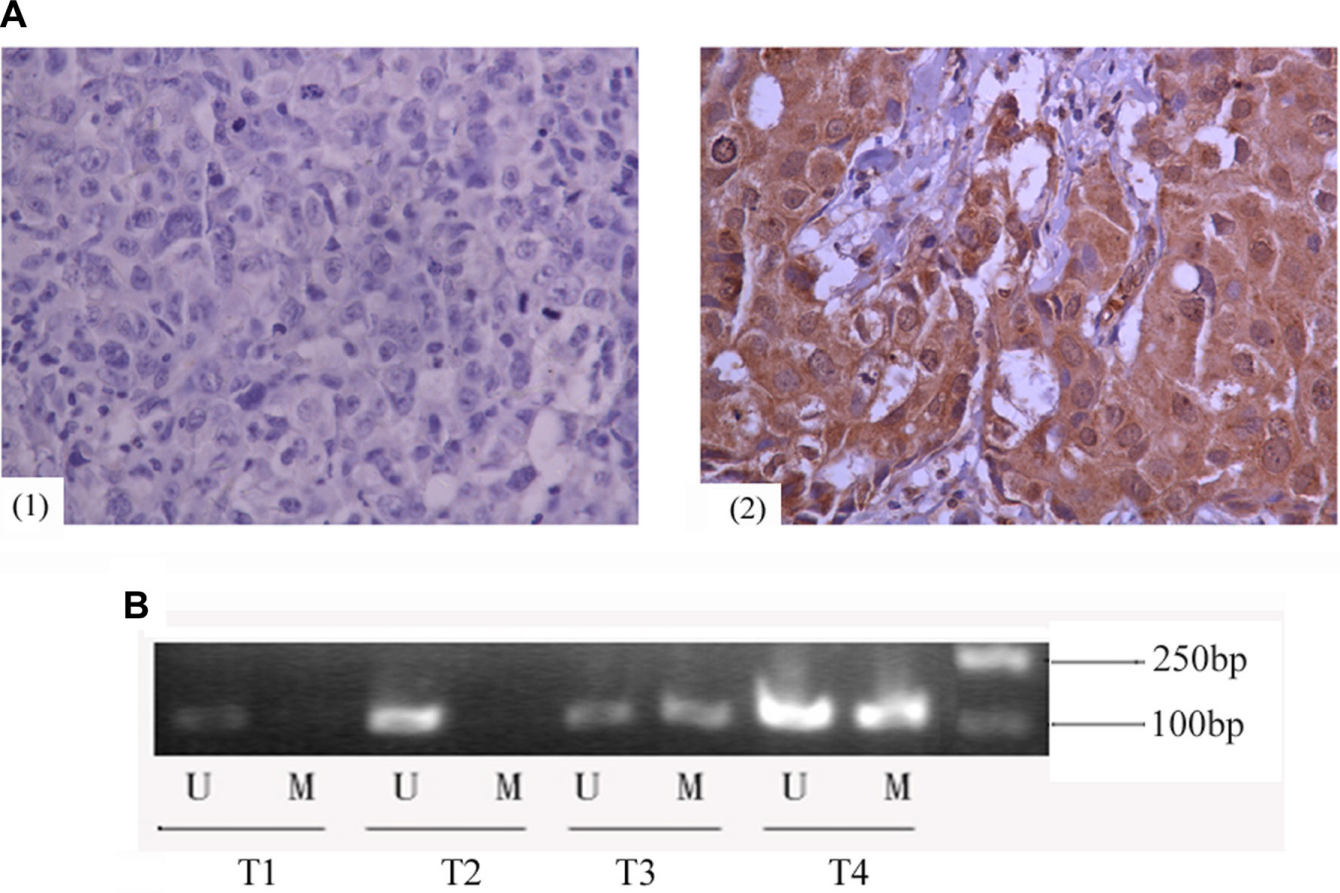
Expression of *GCS* protein and methylation status of *GCS* promoter in invasive ductal breast cancer (**A**) Immunohistochemical analyses of *GCS* protein in invasive ductal breast cancer. Images are representative of two cases that were *GCS*-negative (1) or *GCS*-positive (2), respectively. (**B**) MSP detection of *GCS* promoter methylation in different invasive ductal breast cancer tissues. T1 and T2 were scored as unmethylation, T3 and T4 were scored as methylation. Abrreviations: T, breast cancer tissues; U, unmethylation; M, methylation.

In primary human invasive ductal carcinoma tissues, *GCS* expression was mainly observed in the cytoplasm of cancer cells. Immunohistochemistry analysis revealed GCS-negative and GCS-positive (Figure 1A) expression.MSP was used to measure the methylation status of *GCS*. T1 and T2 are representative of unmethylation status, T3 and T4 are representative of methylation status (Figure 1B). Among 40 cases of *GCS*-positive breast cancer tissues, 25% (10/40) presented a methylated *GCS* promoter. Meanwhile 87.5% (35/40) presented a methylated *GCS* promoter in 40 cases of *GCS*-negative group (Table 1). A significant difference appeared comparing the two groups. This result suggested that methylation of the *GCS* promoter was inversely associated with the *GCS* expression (*r* = −0.63, *p* < 0.01).

**Table 1:** Correlations between *GCS* methylation status and clinicopathological features in invasive ductal breast cancer patients

		*GCS* methylation (45/80, 56.2%)	*GCS* non- methylation (35/80, 43.8%)	*p* value	Pearson correlation coefficient
*GCS* protein	*GCS* (+)	10	30	< 0.001*	−0.630*
	*GCS* (−)	35	5		
Age (years)	< 35	4	1	0.379	−0.132
	≥ 35	41	34		
Tumor stage	T < 5 cm	40	30	0.928	−0.010
	T ≥ 5 cm	5	5		
	N0	20	14	0.690	−0.045
	N1–x	25	21		
Histological stage	I	11	7	0.637	−0.053
	II–III	34	28		
ER	Positive	35	19	0.026*	−0.249*
	Negative	10	16		
PR	Positive	26	15	0.185	0.148
	Negative	19	20		
HER-2	Positive	21	6	0.006*	0.310*
	Negative	24	29		
Ki67	< 14%	14	10	0.806	−0.027
	≥ 14%	31	25		

Note: *Statistical significance (*p* < 0.05).

Abbreviations: ER, estrogen receptor; PR, progesterone receptor; HER-2, human epidermal growth factor-2.

Correlation analysis was also performed between the promoter methylation status and clinicopathological parameters. Compared with the ER negative group of 61.5% (16/26) methylation levels of *GCS* CpG islands, the ER positive group exhibited lower methylation levels of 35.2% (19/54) (*r* = −0.249, *p* = 0.026). Compared with the HER-2 receptor positive group of 77.8% (21/27) methylation levels of *GCS* CpG islands, the HER-2 receptor negative group exhibited lower methylation levels of 45.3% (24/53) (*r* = 0.31, *p* = 0.006). Thus, *GCS* methylation status was negatively correlated with ER positivity, but positively with HER-2 positivity (Table 1). There was no statistical significance in the relationship between *GCS* methylation and other clinicopathological parameters, including age, histological stage, tumor size, nodal stage or Ki67 (Table 1).

### *GCS* promoter methylation correlates negatively with GCS expression in breast cancer cells

**Figure 2:**
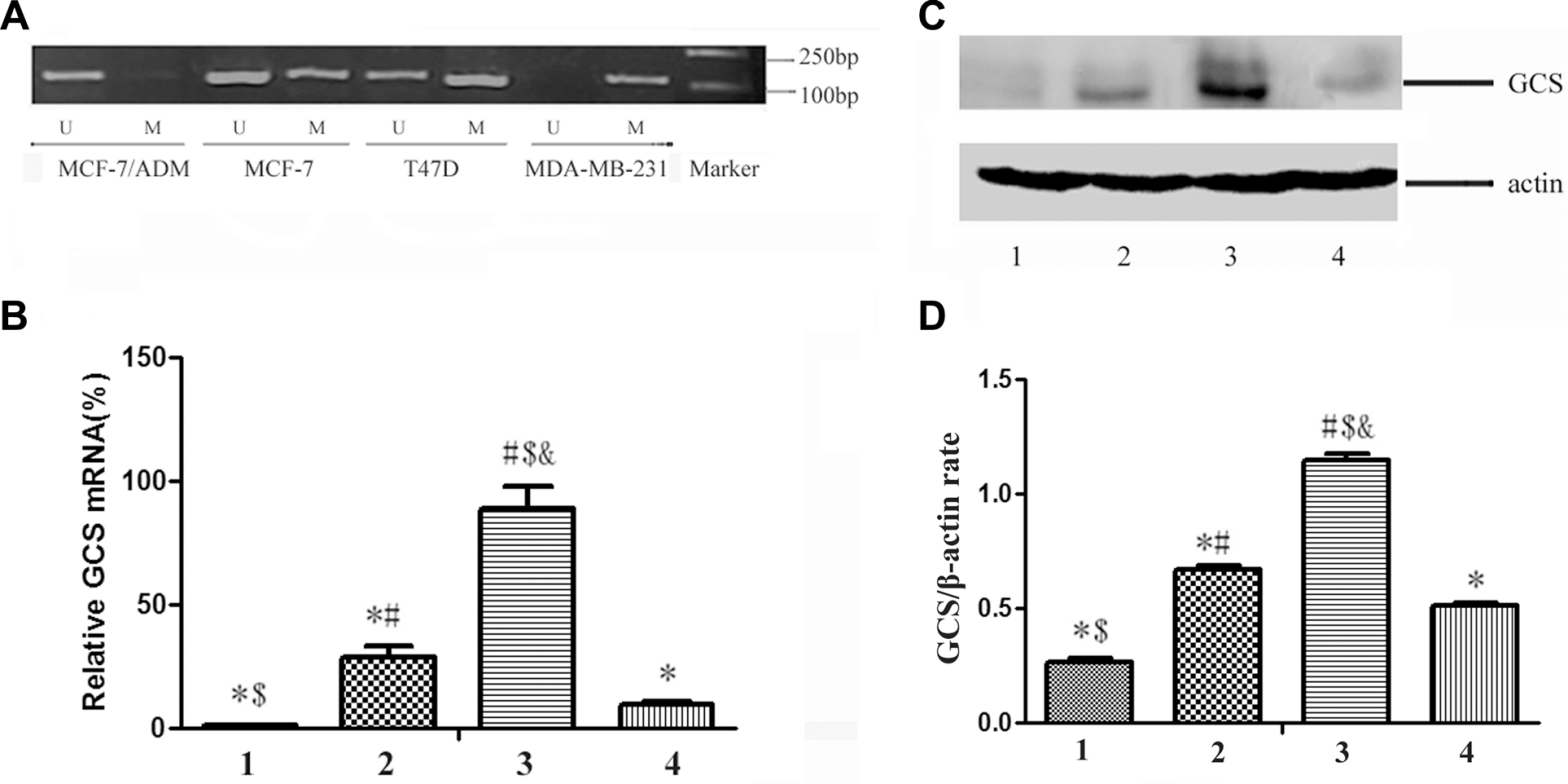
The status of *GCS* promoter methylation and the expression of *GCS* in breast cancer cell lines (**A**) MSP detection of *GCS* promoter methylation. Complete methylation was discovered in MDA-MB-231cell line, partial methylation in MCF-7 and T47D cell lines, but unmethylation in MCF-7/ADM cell line. (**B**) qPCR detection of *GCS* mRNA expression. (**C**) Westernblot detection of *GCS* protein expression. (**D**) Relative expression of GCS protein. The expresssion of GCS in MDA-MB-231 is lowest in the four cell lines both in mRNA level and in protein level; and that in MCF-7/ADM is highest. Notes: 1 represents MDA-MB-231; 2 represents MCF-7; 3 represents MCF-7/ADM; 4 represents T47D ^*^p < 0.05 vs. MCF-7/ADM; ^#^*p* < 0.05 vs. MDA-MB-231; ^$^*p* < 0.05 vs. MCF-7; ^&^*p* < 0.05 vs. T47D.

To explore the possibility that DNA methylation inhibits *GCS*, the methylation status of *GCS* in four human breast cancer cell lines was detected by MSP. Complete methylation was observed in the MDA-MB-231 cell line, partial methylation in MCF-7 and T47D cell lines, but unmethylation in the MCF-7/ADM cell line PCR (Figure 2A).

To evaluate the relationship between different degrees of methylation of the *GCS* promoter and its expression, *GCS* mRNA expression was detected in breast cancer cells by quantitative real-time PCR (Figure 2B). The relative mRNA expression of *GCS* in the MDA-MB-231 cell line was significantly lower than that in the other three cell lines (*p* < 0.05). The relative mRNA expression of *GCS* in the MDR breast cancer cells MCF-7/ADM was significantly higher than that in the other three cell lines (*p* < 0.05). The protein expression of *GCS* was also detected simultaneously in the breast cancer cells by westernblot (Figure 2C, 2D); the results coincided with the mRNA expression.

### Reversal of the methylation status in breast cancer cells by 5-Aza-dc

**Figure 3:**
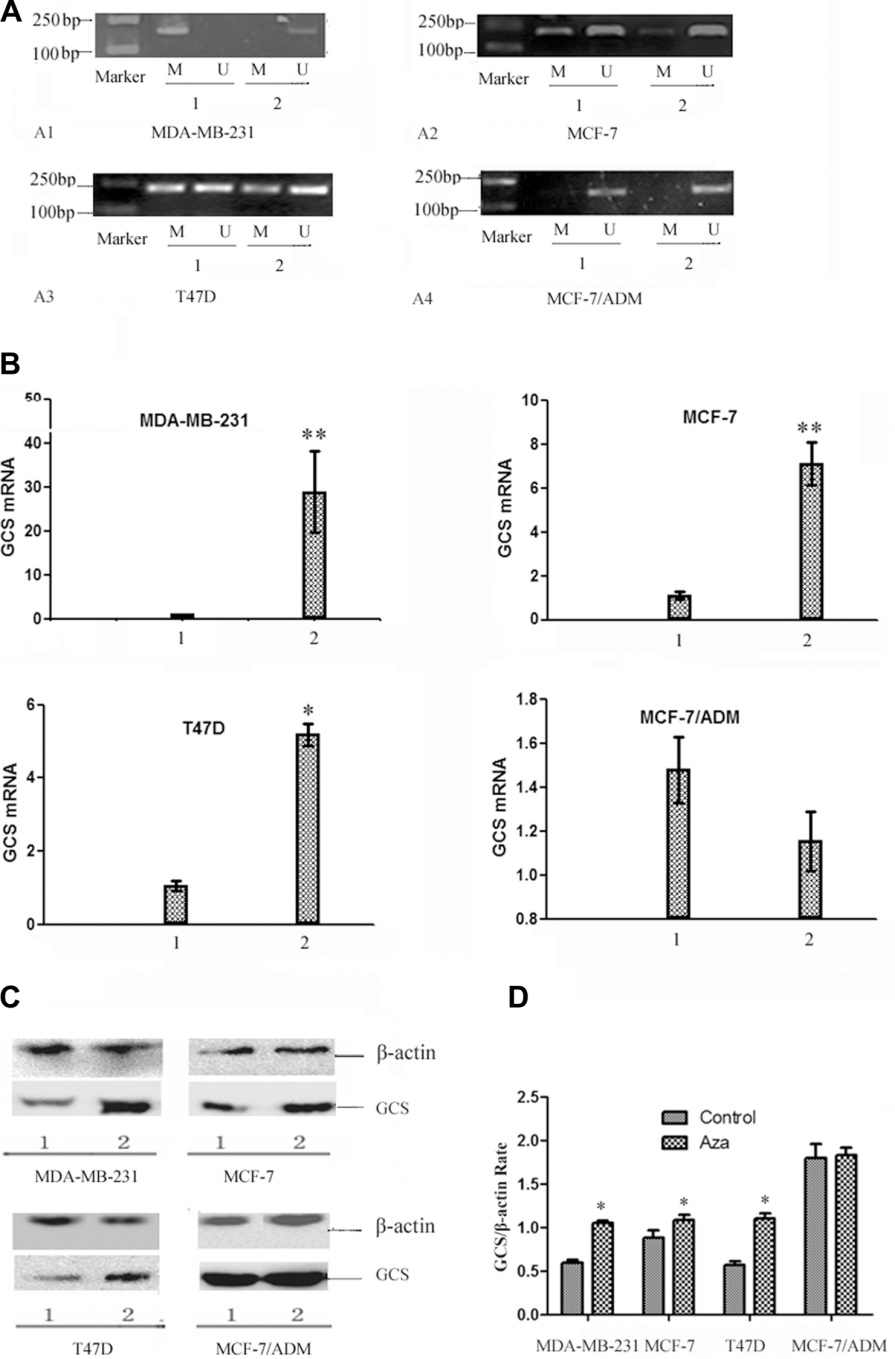
The changes of the status of *GCS* promoter methylation and the expression of *GCS* in breast cancer cell lines by 5-Aza-dc (**A**) MSP analysis of *GCS* methylation status before and after 5-Aza-dc treatment. After treated with 5-Aza-dc, MDA-MB-231 changed from methylation into unmythylation, meanwhile there was no significant change in MCF-7/ADM. In MCF-7 and T47D cells, methylated bands were still present, but became increasingly weaker. MDA-MB-231 (A1); MCF-7 (A2); T47D (A3); MCF-7/ADM (A4). Notes: 1 represents before treatment; 2 represents after treatment. (**B**) qPCR analysis of *GCS* mRNA expression before and after 5-Aza-dc treatment. qPCR was used to detect the alteration of *GCS* mRNA expression. The expression of *GCS* mRNA increased significantly in three cell lines except MCF-7/ADM after treated with 5-Aza-dc. Notes: 1 represents before treatment; 2 represents after treatment. ^*^p < 0.05 vs 1; ^**^p < 0.01vs 1; (**C**) Westernblot analysis of GCS protein expression before and after 5-Aza-dc treatment. The expression of *GCS* protein increased significantly in three cell lines except MCF-7/ADM after treated with 5-Aza-dc, which in coordiance with that of the *GCS* mRNA expression. Notes: 1 represents before treatment; 2 represents after treatment. (**D**) Relative expression of GCS protein. Notes: ^*^p < 0.05 vs control.

The high correlation between *GCS* promoter methylation and lack of gene expression prompted us to further explore the role of epigenetics in *GCS* gene expression. Previous reports demonstrated that treatment with the demethylating agent 5-Aza-dC, a DNA methyltransferases (DNMTs) inhibitor, could restore silenced gene expression. Thus, we explored the responsiveness of the four breast cancer cell lines to 5-Aza-dC. In MDA-MB-231 cells, methylated *GCS* gene bands disappeared and non-methylated *GCS* bands appeared (Figure 3A–3A1). In MCF-7 and T47D cells, methylated bands were still present, but became increasingly weaker (Figure 3A–3A2, 3A–3A3). However, no change was found in MCF-7/ADM cells (Figure 3A–3A4).

Treatment with 5-Aza-dC resulted in a significant enhancement in *GCS* mRNA (Figure 3B) and protein expression (Figure 3C, 3D), relative to untreated cells, in MDA-MB-231 and MCF-7, T47D cells (*p* < 0.05). However, in the corresponding MDR cell line MCF-7/ADM, which initially displayed substantial *GCS* overexpression, there was no significant change in *GCS* gene expression before or after 5-Aza-dc treatment (*p* > 0.05).

### The changes of drug resistance in breast cancer cells by 5-Aza-dc

**Figure 4:**
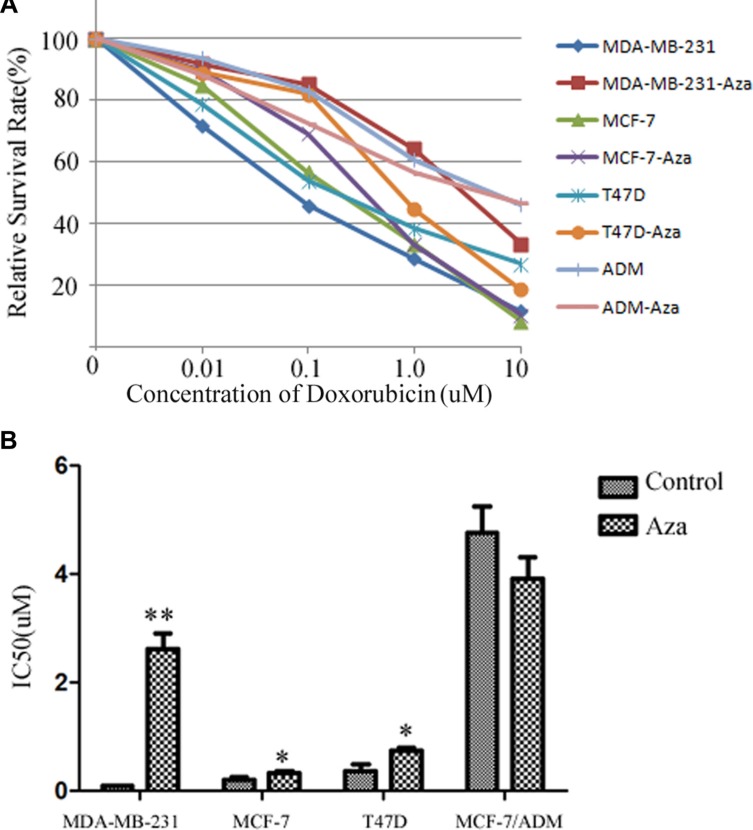
The changes of drug resistance in breast cancer cells by 5-Aza-dc (**A**) The survival curve of the cell lines after added to different concentration of doxorubicin. All cell lines with or without 5-Aza-dc for 48 h, then the cells were seeded in 96-cells plate. Different concentration of doxorubicin were added for 24 h, then MTS was adopted to analysize the survival rate. Dose-response curves were plotted from three independent experiments. (**B**) The IC50 of each group. The relative drug resistance was determined by comparing the IC50 (drug concentration causing 50% inhibition of cell growth) from growth inhibition curves. Notes: ^**^p < 0.01 vs. control; ^*^p < 0.05 vs control.

Doxorubicin is a common drug for many carcinomas. The results demonstrated that IC50 for doxorubicin significantly increased from 0.089 ± 0.002 μmol/L to 2.678 ± 0.267 μmol/L in MDA-MB-231 cells after treated with 5-Aza-dc (*p* < 0.01). In MCF-7 and T47D cells, the IC50 also increased (*p* < 0.05). However, there was no significant change in the cell line MCF-7/ADM (Figure 4A, 4B).

### Expression of DNMT1 and DNMT3a protein in breast cancer cells

**Figure 5:**
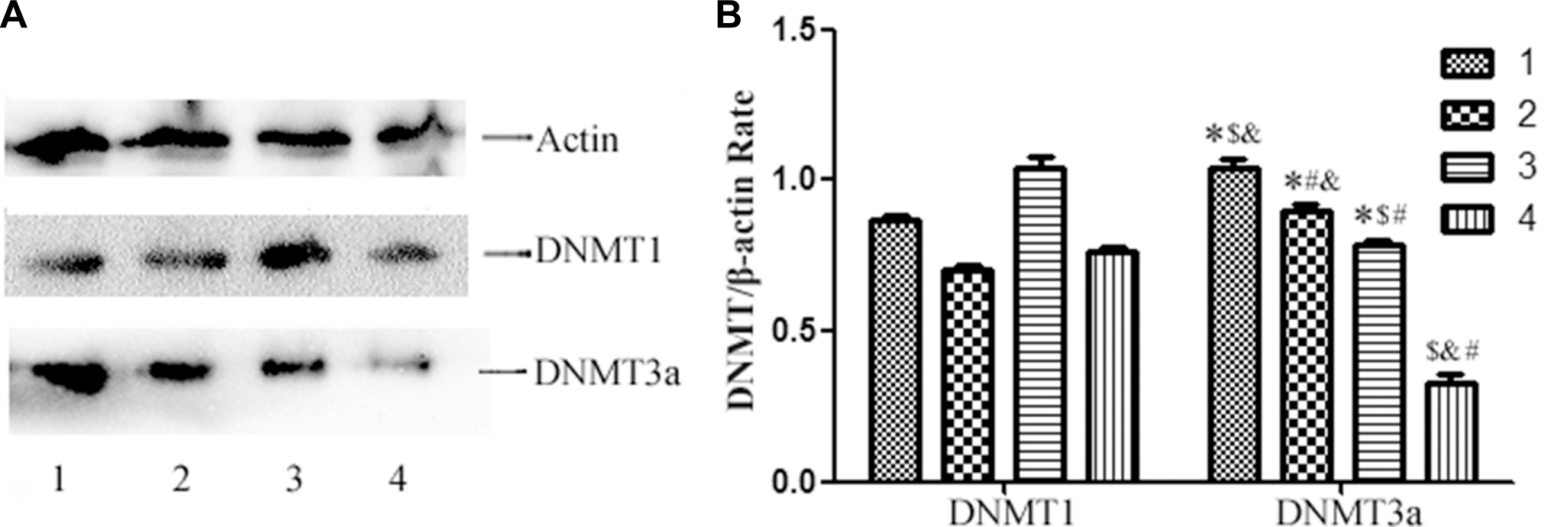
Westernblot analysis of DNMT1 and DNMT3a protein expression in the cell lines (**A**) Westernblot analysis of DNMT1 and DNMT3a protein expression. All the cell lines express DNMT1protein and DNMT3a protein, however the expression of DNMT3a protein is highest in MDA-MB-231 and that is lowest in MCF-7/ADM. (**B**) Relative DNMT1 and DNMT3a protein expression in each cell line. Notes: 1 represents MDA-MB-231; 2 represents MCF-7 ; 3 represents T47D; 4 represents MCF-7/ADM ^*^p < 0.05 vs. MCF-7/ADM; ^#^*p* < 0.05 vs. MDA-MB-231; ^$^*p* < 0.05 vs. MCF-7; ^&^*p* < 0.05 vs. T47D.

In order to detect the mechanisms of methylation of GCS promoter, we analyzed the expression of DNMT1and DNMT3a protein in each cell line. The results displayed that all the cells expressed the DNMT1 and DNMT3a protein. The expression of DNMT1 was no significant difference. However the expression of DNMT3a protein is highest in MDA-MB-231and that is lowest in MCF-7/ADM (Figure 5A, 5B).

## DISCUSSION

The development of a malignant disease occurs via a multistage process, including genetic and epigenetic modifications. Epigenetics is a kind of inheritable gene expression mechanism that does not change the DNA sequence, and involves DNA methylation, histone acetylation and chromatin remodeling [20]. DNA methylation is important in various biological processes, such as genomic imprinting, inactivation of X chromosomes, cell differentiation and development [21]. Increasing research has focused on the relationship between DNA methylation and MDR. DNA methylation is far more vulnerable than the DNA sequence to external factors. DNA methylation changes can occur rapidly, resulting in resistance arising quickly following chemotherapy treatment [22, 23]. Demethylation of CpG islands in the *MDR1* promoter region is a mechanism of chemoresistance, which induces the expression of P-gp and the MDR phenotype [24].

Intensive investigations have been performed regarding correlations between protein expression of *GCS* and MDR [9, 10, 11]. However, the mechanism of protein expression by promoter methylation of *GCS* is not completely understood. To date, most studies have focused on the association with *GCS* and its downstream effectors. And many results have been obtained. Gouaze et al. [25] suggested that *GCS* blockade resensitizes MDR breast cancer cells to anticancer drugs via downregulation of *MDR1*. Liu et al. [26] further demonstrated that *GCS* upregulates *MDR1* expressions through cSrc and beta-catein signaling. Zhang et al. showed that *GCS* can increase the expression of *MDR1* through NF-κ B signaling in K562/AO2 cells [27]. No relevant research on upstream effectors that regulate *GCS* expression has been reported. The *GCS* promoter also contains a potential CpG island; thus, epigenetic changes might regulate its expression. The purpose of the present study was to determine whether epigenetic changes influence*GCS* expression in breast cancer. To reach this aim, we used MSP to map the methylation status of CpG sites in the *GCS* promoter and then analyzed its association with *GCS* expression. The results showed that in breast cancer tissues, DNA methylation could inhibit *GCS* expression. Methylation of the *GCS* promoter was inversely associated with *GCS* expression. The conclusions were similar to a previous investigation: Lincke et al. demonstrated a rough correlation between hypomethylation and transcription of the 5′end of the *MDR1* and *MDR3* genes [28]. Hirofumi et al. suggested that methylation status of *BCRP* was inversely correlated with its expression in lung cancer cells [29], multiple myeloma [30] and pancreatic cancer [31].

We also analyzed the correlation between methylation status of the *GCS* promoter and clinicopathological parameters. The results indicated that methylation of the *GCS* promoter was negatively associated with ER positivity, but positively associated with HER-2 positivity. The results were consistent with our previous study, in which the expression of *GCS* in invasive ductal breast cancer correlated with high ER and low HER-2 status [32]. Although the study of Liu demonstrated that GCS overexpression is highly associated with ER-positive and HER-2-positive breast cancers that have metastasized, this was a small study [33]. And the correlations between *GCS* promoter methylation and ER or HER-2 status need to be further investigated in future studies. *GCS* methylation was not correlated with tumor size, lymph metastasis or histological stage in this study, suggesting that *GCS* methylation would not be a good prognostic indicator for breast cancer.

The presence of a methylated sequence in the 5′regulatory regions of certain genes appears to determine the level of transcription [34], and DNA methylation often induces gene inactivation in *in vitro* transcription assays [35]. In a recent publication, hypermethylation of CpG dinucleotides in the *MDR1* promoter region also contributed strongly to differences in gene expression in related cell lines [15]. To investigate whether certain distinct DNA methylation patterns were associated with the *GCS* phenotype of breast cancer cells, we analyzed the methylation status and the expression of *GCS* by MSP, qPCR and westernblot. We observed that DNA methylation existed in breast cancer cells, and that methylation of *GCS* repressed the gene expression. We examined the methylation status of the *GCS* promoter region in four breast cancer cell lines that differed in their respective *GCS* expressions. We found that the promoter of very low level expressing cells was almost completely methylated, whereas high and medium *GCS* expressions were either completely or almost completely unmethylated. The results clearly indicated an inverse correlation between methylation status and *GCS* gene expression in breast cancer cells.

CpG island hypermethylation and consequent gene silencing in cancer was found to be induced by the deregulation of DNMTs [36]. The DNMT inhibitor, 5-Aza-dc, could reactivate silenced genes and has become a relevant molecular therapy, currently used to treat hematological malignancies [37, 38]. To further explore the relationships among DNA methylation, *GCS* expression and MDR, 5-Aza-dc was used to treat breast cancer cells. 5-Aza-dc could reverse *GCS* promoter methylation and induced re-expression at the messenger RNA and protein levels in MDA-MB-231, MCF-7 and T47D cell lines.

The correlation between GCS CpG islands methylation and chemotherapy drug sensitivity was assessed by MTS. The IC50 value of MDA-MB-231, MCF-7 and T47D increased significantly after treated with 5-Aza-dc. This suggested that demethylation of *GCS* resulted in an apparent increase in the generation of multidrug-resistant clones. No change was found in MCF-7/ADM cell lines, which suggested that restoration of *GCS* gene expression was caused by transcriptional upregulation rather than by changed *GCS* mRNA stability. In order to explore the mechanisms of GCS methylation, we detected the expression of DNMT1 and DNMT3a of the four cell lines by western blot, we can see that the DNMT1 protein of the four cell lines didn't have significant differences, but the expression of DNMT3a protein is highest in MDA-MB-231and that is lowest in MCF-7/ADM. From the result, we can deduce that GCS methylation is related to the expression of DNMT3a.

These findings suggested that promoter methylation is responsible for transcriptional silencing of GCS in patient plasma cells and in cell lines. Demethylation of the promoter was necessary for *GCS* re-expression and for *GCS*-induced MDR. CpG island methylation can cause repression of gene expression either directly through transcription factors [39] or indirectly through recruitment methyl-binding proteins [40, 41]. Whether recruitment of methyl-binding proteins is involved in the case of the *GCS* gene remains to be determined. The mechanism by which DNA methylation controls gene expression in this model requires further evaluation and a more detailed understanding of the molecular basis of the MDR phenotype may provide further opportunities for subsequent clinical intervention.

## MATERIALS AND METHODS

### Clinical samples

Tissue samples from 150 patients with primary invasive ductal breast carcinoma who underwent complete dissection of the breast and axillary lymph nodes were collected at the Yuhuangding Hospital affiliated to Qingdao University, China, between Jan 2011 and Jun 2012. No patients had preoperative chemotherapy and informed consent for pathological evaluation was obtained from all patients prior to surgery. Then immunohistochemical analyses were adopted to detect the expression of *GCS* protein and the methods will be described in Immunohistochemical analyses. Then 40 cases *GCS*-positive and 40 cases *GCS*-negative cases were selected for our following research.

Patient and tumor characteristics of the 40 *GCS*-positive and 40 *GCS*-negative cases are summarized in Table 2. The use of these tissues was approved by the Research Ethics Committee of Yuhuangding Hospital, and we obtained informed written consent for pathological evaluation from all participants involved in our study.

**Table 2:** Clinicopathological features of patients with invasive ductal breast cancer

A. Clinicopathological features of GCS positive invasive ductal breast cancer patients		
Characteristics	Number	%
Age (years)		
< 35	1	(2.50)
≥ 35	39	(97.5)
Tumor size		
T < 5 cm	36	(90.0)
T ≥ 5 cm	4	(10.0)
Nodal stage		
N0	14	(35.0)
N1–x	26	(65.0)
Histological stage		
I	7	(17.5)
II	18	(45.0)
III	15	(37.5)

B. Clinicopathological features of GCS negative invasive ductal breast cancer patients		
Characteristics	Number	%
Age (years)		
< 35	4	(10.0)
≥ 35	36	(90.0)
Tumor size		
T < 5 cm	34	(85.0)
T ≥ 5 cm	6	(15.0)
Nodal stage		
N0	20	(50.0)
N1–x	20	(50.0)
Histological stage		
I	11	(27.5)
II	20	(50.0)
III	9	(22.5)

### Ethics statement

The work was conducted in accordance with the Declaration of Helsinki. Informed consent was obtained from all the patients in this study. All patients signed the informed consent for use of specimens, and the study was approved by the Institutional Review Board (Medical Ethics Committee of Yuhuangding Hospital).

### Cell culture

Three drug-sensitive breast cancer cell lines, MCF-7 (ER-positive, PR-positive, HER-2-negative), MDA-MB-231 (triple-negative) and T47D (ER-positive, PR-positive, HER-2-negative), were obtained from the American National Cancer Institute. The multidrug-resistant breast cancer cell line, MCF-7/ADM, was selected from MCF-7 with doxorubicin treatment in stages [6]. MCF-7, MDA-MB-231 and MCF-7/ADM were maintained in RPMI 1640 (Gibco, USA) medium supplemented with 10% (v/v) fetal bovine serum (FBS), 100 U/ml penicillin and 100 μg/ml streptomycin and T47D were maintained in L-15(Gibco, USA). All cells were cultured in a humidified atmosphere containing 5% CO_2_ at 37°C.

### Immunohistochemical analyses

Immunohistochemical staining was carried out using the DAKO Envision detection kit (Dako, Carpinteria, CA, USA). In brief, paraffin-embedded tissue blocks were sectioned (4 μm-thick), dried, deparaffinized and rehydrated. Antigen retrieval was performed in a microwave oven for 15 min in 10 mM citrate buffer (pH 6.0). Then cells were embedded in 4% neutral formaldehyde for 2 h. For all samples, endogenous peroxidase activity was blocked with a 3% H_2_O_2_-methanol solution. The slides were blocked with 10% normal goat serum for 10 min and incubated with an appropriately diluted primary antibody overnight at 4°C. The slides were then probed with an HRP-labeled polymer conjugated to an appropriate secondary antibody for 30 min. The antibodies against estrogen receptor (ER, Product No E07165), progesterone receptor (PR, Product No E06575), HER-2 (No E07758) and Ki67 (Product No E07806) were purchased from Roche, Switherland and are all work fluid and the *GCS* antibody was purchased from Bioss, Beijing, China (diluted 1:300, Product No bs-0701P).

Staining results were interpreted by two breast pathologists who were blinded to patient outcomes. Tumors with 1% or more positively stained nuclei were considered positive for ER and PR expression. Ki67 staining was determined to be positive when more than 14% of the nuclei were stained [32, 42]. HER-2 was scored by counting the number of positively stained cells on the membrane and expressed as a percentage of total tumor cells according to the American Society of Clinical Oncology (ASCO) and the College of American Pathologists (CAP) guidelines using the following categories: 0, no immunostaining; 1+, weak, incomplete membranous staining in any proportion of tumor cells; 2+, complete membranous staining, either non-uniform or weak in at least 10% of tumor cells; and 3+, uniform, intense membranous staining in > 10% of tumor cells. HER-2 results were considered positive in cases with 3 + membranous staining of IHC or gene amplification by fluorescence *in-situ* hybridization (FISH) irrespective of IHC results using the diagnostic criteria described [43].

A dual semi-quantitative scale combining staining intensity and percentage of positive cells was used to evaluate *GCS* protein staining. The staining intensity of the cell plasma was scored as 0 (negative), 1 (weak), 2 (moderate) or 3 (strong). The percentage of positive cells was scored as follows: 0, no staining or staining in < 5% of tumor cells; 1, staining in 5% to 25% of cells; 2, staining in 26% to 50% of cells; 3, staining in 51% to 75% of cells; 4, staining in > 75% of cells. For *GCS*, cytoplasmic staining was considered positive with an immunohistochemical score ≥ 2, or negative with an immunohistochemical score < 2 [32].

### DNA extraction and MSP

Genomic DNA from 40 paired *GCS*-positive and *GCS*-negative cases of primary invasive ductal breast carcinoma patients and four breast cancer cell lines, was isolated by proteinase K method. The genomic DNA was modified with the CpGenome^TM^ Direct Prep Bisulfite Modification kit (Millipore, USA), according to the manufacturer's instructions. MSP primers were designed online (http://www.urogene.org/methprimer/index1.html). Primers were synthesized (Sangon, Shanghai, China) to detect bisulfate-induced changes affecting methylated (M) and unmethylated (U) alleles. The MSP primer sequences were as follows: 5′-TTTTGGTTAATAAGGT GAATTTCG-3′ (MF), 5′-AACCGAACTACGAACTACAATAAC G -3′ (MR) and 5′-GGTT AATAAGGTGAAATTTTGTGCT -3′ (UF), 5′-CCAAACTACAAACTACAAT AACACA-3′ (UR). The size of the non-methylated PCR product was 185 bp and the methylated PCR product was 187 bp. Each MSP reaction included approximately 100 ng of bisulfite-treated DNA, 25 pmoles of each primer, 100 pmoles dNTPs, 2.5 μl 10 × PCR buffer and 1 unit of Taq Polymerase (Invitrogen, Carlsbad, CA, USA) in a final reaction volume of 25 μl. The PCR reaction was as follows: initial denaturation for 5 min at 94°C; followed by 35 cycles of denaturation for 30s at 94°C, primer annealing for 30s at 60°C, and polymerization for 30s at 72°C; and final extension for 10 min at 72°C. MSP products were analyzed by 2% agarose gel electrophoresis stained with ethidium bromide. In invasive ductal breast cancer, the MSP products in the M lanes were scored as methylation, and those in the U lanes were scored as unmethylation [44, 45]. In breast cancer cells, cells were scored as unmethylation when bands were present only in the unmethylated DNA lane and as complete methylation when bands were present in the methylated DNA lane. Bands present both methylated and unmethylated lanes were scored as partial methylation [46].

### RNA extraction and quantitative real-time PCR (qPCR)

Total RNA was isolated using the Trizol-Reagent (Invitrogen) as recommended by the manufacturer, and quantitative real-time PCR was used to detect *GCS* mRNA. qPCR was performed using a SYBR Green Real-time PCR MasterMix (TOYOBO, Japan). The primers for *GCS* were as follows : sense: 5-CCTT TCCTCTCCCCACCTTCCTCT-3′, antisense: 5′-GGTT TCAGAAGAGAGACACCTGGG-3′ [47]. The expression of the β-actin (sense: 5′-ACCCCCACTGAAAAAG ATGA-3′, antisense: 5′-ATCTTCAAACCTCCATGA TG-3′) gene was used as an internal control set. The final volume was 25 μl, and an iCycler iQ Real-Time PCR Detection System (Bio-Rad) was used for qPCR. The qPCR reaction was as follows: initial denaturation for 5 min at 94°C; followed by 35 cycles of denaturation for 30s at 94°C, primer annealing for 30s at 60°C, and polymerization for 30s at 72°C; and final extension for 10 min at 72°C. The relative mRNA expressions were calculated using the 2^−ΔΔ^Cq method, where ^ΔΔ^Cq = target Cq - control Cq;^ΔΔ^Cq = ^Δ^Cq target - ^Δ^Cq calibrator (Cq, cycle threshold).

### Western blot

Cells were washed with phosphate buffered saline and lysed in 100 μl of lysis buffer (10 mM Tris-HCl, pH 7.4, 5 mM MgCl2, 1 mM EDTA, 25 mM NaF, fresh 100 μM Na_3_VO_4_ and l mM dithiothreitol). Cell lysates were centrifuged for 10 min at 14,000 × g. Using a previously described method [48], equal amounts of protein (100 μg) were resolved on a 10% SDS-polyacrylamide gel and transferred electrophoretically to a polyvinylidene fluoride membrane. The membranes were blocked with fat-free milk (5%) in Tris-Buffered Saline and Tween 20 (10 mM Tris-HCl, pH 7.4, 150 mM NaCl, 0.1% Tween-20) at room temperature for 2 h. The membrane was immunoblotted with rabbit polyclonal against human *GCS* antibody (Santa Cruz, USA, diluted 1;1,000) in 5% fat-free milk in Tris-Buffered Saline and Tween 20. As a control for equivalent protein loading, the filters were simultaneously incubated with mouse monoclonal antibody against human β-actin (diluted 1:1,000). Detection was performed using enhanced chemiluminescence (Millipore, USA). All analyses were performed in triplicate in three separate experiments.

### 5-aza-2′-deoxycytidine (5-Aza-dc) treatment

Breast cancer cell lines (MCF-7, MDA-MB-231, T47D and MCF-7/ADM) were split into low density (30% confluence) 12 h before treatment. Cells were treated with 5-Aza-dc (Sigma, St. Louis, MO, USA) at a concentration of 5 μM for 72 h. At the end of treatment, RNA was extracted from the cells as described above.

### Cytotoxicity assay for cell survival (MTS)

The MTS assay was used to assess the effect of methylation of *GCS* promoter on the chemosensitivity of breast cancer cells to anticancer drug [49]. In brief, cells were plated in a 96-well plate at a density of 5 × 10^3^ cells per well for 24 h, and then incubated with different concentrations of doxorubicin (Dalian Meilunbio Co., Ltd., China) for 24 h. Then 20 μl of MTS was added to each well and incubated at 37°C for 3 h. Optical densities (ODs) were detected using a spectrometric absorbance of 570 nm against a background of 630 nm on a Bio-Rad microplate reader (Hercules, CA, USA). The value of (A570 anticancer drug +/A570 anticancer drug−) × 100% indicated cell viability. Dose-response curves were plotted from three independent experiments. The relative drug resistance was determined by comparing the IC50 (drug concentration causing 50% inhibition of cell growth) from growth inhibition curves.

### Statistical analysis

All calculations were performed using the SPSS16.0 for windows statistical software package (SPSS, Chicago, IL, USA). Chi-squared or Fisher's exact tests, and Pearson correlation coefficient, were used to analyze the relationship between the expression of *GCS* and each histopathological variable. Cellular data were presented as the mean ± standard deviation. One-way ANOVA and Dunnett's T3 tests were used to determine statistical significance. *P*-values less than 0.05 were considered statistically significant.
